# Rosemary Extract as a Potential Anti-Hyperglycemic Agent: Current Evidence and Future Perspectives

**DOI:** 10.3390/nu9090968

**Published:** 2017-09-01

**Authors:** Madina Naimi, Filip Vlavcheski, Hesham Shamshoum, Evangelia Tsiani

**Affiliations:** 1 Department of Health Sciences, Brock University, St. Catharines, ON L2S 3A1, Canada; madinanaimi@gmail.com (M.N.); fv11vi@brocku.ca (F.V.); hs12af@brocku.ca (H.S.); 2 Centre for Bone and Muscle Health, Brock University, St. Catharines, ON L2S 3A1, Canada

**Keywords:** insulin resistance, diabetes, rosemary extract, rosmarinic acid, carnosic acid, carnosol

## Abstract

Type 2 diabetes mellitus (T2DM), a disease on the rise and with huge economic burden to health care systems around the globe, results from defects in insulin action (termed insulin resistance) combined with impaired insulin secretion. Current methods of prevention and treatments for insulin resistance and T2DM are lacking in number and efficacy and, therefore, there is a need for new preventative measures and targeted therapies. In recent years, chemicals found in plants/herbs have attracted attention for their use as functional foods or nutraceuticals for preventing and treating insulin resistance and T2DM. Rosemary is an evergreen shrub indigenous to the Mediterranean region and South America, which contains various polyphenols. Rosemary extract and its polyphenolic constituents have been reported to have antioxidant, anti-inflammatory, anticancer, and anti-hyperglycemic properties. The current review summarizes the existing in vitro and in vivo studies examining the anti-diabetic effects of rosemary extract and its polyphenolic components and highlights the known mechanism of action.

## 1. Introduction

Type 2 diabetes mellitus (T2DM) is a disease resulting from impairments in insulin action and insulin secretion [1]. Diminished insulin action and insulin secretion lead to deregulation of glucose and fat metabolism in various tissues including skeletal muscle, adipose tissue and liver and failure to maintain normal blood glucose levels. This prolonged pathological state characterized by hyperglycemia and hyperlipidemia leads to complications such as retinopathy; neuropathy; nephropathy; and microvascular, macrovascular and cardiovascular problems. According to the World Health Organization and the International Diabetes Federation (IDF) estimates, T2DM is rapidly growing and will be affecting more than 439 million adults by 2030 [2]. At present, there is no cure for diabetes mellitus as is true for many of the major chronic diseases inflicting the world’s population at large. Current therapeutic strategies for diabetes mellitus are aimed at management and alleviation of the underlying pathological processes and include lifestyle modifications such as healthy diet, weight management and regular physical activity coupled with medication/drug interventions [3].

**Figure 1:**
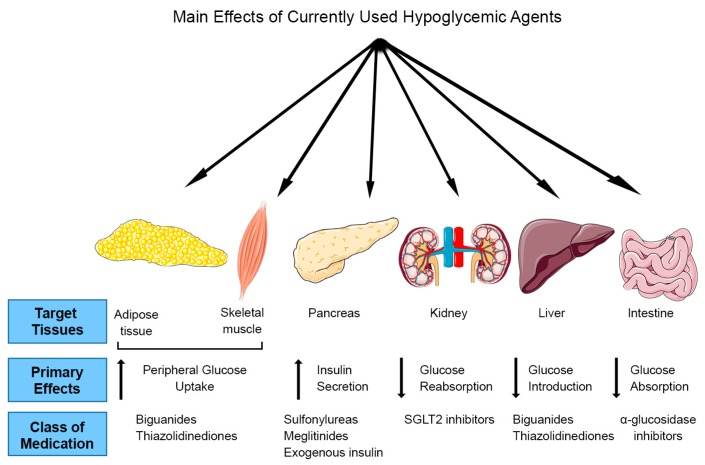
Target tissues and mechanism of action of current anti-diabetic drugs.

Oral medications for T2DM treatment include drugs that stimulate insulin release from β-cells (sulfonylureas and meglitinides); inhibit intestinal glucose absorption (α-glucosidase-inhibitors); increase peripheral glucose transport; and reduce hepatic glucose output through inhibition of gluconeogenesis (biguanides and thiazolidinediones) (Figure 1). Metformin (biguanide) is the most widely used treatment for T2DM; unfortunately, it is associated with increased lactic acid acidosis [4] and 30% of patients report gastrointestinal problems such as diarrhea, cramps, nausea and vomiting that can lead to discontinuation of the drug [5]. In the last decade, several new drugs for T2DM were introduced. Dipeptidyl peptide 4 (DDP-4) inhibitors or gliptins (sitagliptin, saxagliptin, and linagliptin) inhibit glucagon release resulting in reduced blood glucose levels, however significant side effects include increased risk of heart failure, pancreatitis and pancreatic cancer [6]. Moreover, glucagon-like peptide 1 (GLP-1) receptor agonists (lxenatide and liraglutide) stimulate insulin and inhibit glucagon release thereby lowering the blood glucose levels. Although GLP-1 agonists have lower risk of hypoglycemia compared to sulfonylureas and meglitinides, they work in the same pathway as DPP-4 inhibitors and there are concerns of an increased risk of pancreatitis and pancreatic cancer [7]. Oral sodium-glucose cotransporter 2 (SGLT2) inhibitors (canagliflozin and dapagliflozin) are the newest anti-diabetic drugs, and work by endorsing urinary glucose excretion and inhibiting renal glucose reabsorption. Although they show promising results for T2DM therapy, their side-effects may include severe urinary tract infections, ketoacidosis and hypotension [8,9].

Overall, there is a continued interest to find more effective drugs with less side effects. On the other hand, despite the availability of various compounds to manage T2DM, the disease is a global health problem with 50% of the affected individuals living in poverty stricken areas in Africa and Asia and therefore there is an urgent need to find effective but less expensive medicine. The study of novel compounds that can exert an insulin-like effect, increase insulin sensitivity and even improve efficacy of existing medication at lower doses and reduce their adverse effects is highly desired and they will widen the spectrum of preventative and treatment options for insulin resistance and T2DM.

The use of plants for healing purposes predates recording history and forms the origin of much of modern medicine [10]. Phytotherapy is still the mainstay of about 75–80% of the world’s population centralized in developing countries for primary health care. Notably, in the last few years, there has been a major rise in phytotherapy in the developed world. In Germany and France, many herbs and herbal extracts are used as prescription drugs and their sales in the countries of the European Union were around 6 billion in 1991 and might be over 20 billion dollars now [10,11].

Many conventional drugs originate from plant sources including aspirin (from willow tree bark), digoxin (from foxglove), quinine (from cinchona bark), and morphine (from the opium poppy). Prior to and after the discovery of insulin, herbs with hypoglycemic effects have been used in folk medicine and are still prevalent. As evidence of this concept, metformin, which is the first line of treatment for T2DM, was purified from the French lilac *Galega officinalis* L.

The development of drugs from plants continues, with drug companies engaged in large-scale pharmacologic screening of various plants including herbs. Any part of a plant, including leaves, stems, flowers, roots, and seeds can be formulated as raw material or extracts. Maceration of plants with water, alcohol or other solvents results in extraction of their active pharmaceutical ingredients including fatty acids, sterols, alkaloids, polyphenols, glycosides, saponins, and others [10]. Recently, a web server (http://bio-hpc.eu/dia-db) has been established that can be used to predict diabetes-related bioactive compounds. The prediction is accomplished by: (1) inverse virtual screening of the input molecule chosen by the user against a set of protein targets identified as key elements in diabetes; and (2) by comparing it to the database of anti-diabetic drugs. This website could be utilized in plant extracts to explore the anti-diabetic potential of specific phytochemical contents of the extract.

## 2. Phenolic Compounds of Rosemary

Rosemary (*Rosmarinus officinalis* L.) is an aromatic, evergreen shrub plant belonging to the labiatae family (Lamiaceae) and indigenous to the Mediterranean region and South America [12,13,14,15,16]. The fresh and dried leaves have been extensively used as seasoning as well as in traditional medicine [16]. Historically, rosemary has been used medicinally to treat renal colic and dysmenorrhea, stimulate hair growth and relieve symptoms caused by respiratory disorders [12,14,17]. Today, rosemary extract is often used in aromatherapy to treat anxiety-related conditions and increase alertness [15,17].

Formulations of rosemary include the raw leaves, and extracts of rosemary. Conventional extraction methods such as hydrodistillation, decoction and maceration along with other methods including pressurized liquid extract, enzyme-assisted extraction, nanofiltration and solid-phase extraction have been employed to extract polyphenols from plant materials [10,18].

Rosemary extract (RE) contains different classes of polyphenols including phenolic acids, flavonoids and phenolic terpenes [18,19,20,21,22] (Table 1). Phenolic acids include: (a) the hydroxycinnamic acids: rosmarinic acid, chlorogenic acid, p-coumaric acid, m-hydroxybenzoic acid, coumaroylquinic acid, ferulic acid and coumaric acid; (b) the hydroxybenzoic acids: vanillic acid, syringic acid, caffeic acid, protocatechuic acid, dicaffeoylquinic; acid and (c) the hydroxyphenylacetic acids: homovanillic acid and p-hydroxybenzoic acid. Flavonoids include the flavones: apigenin, luteolin, hispidulin and genkwanin. In addition, they include the flavonols: rutin, kaempferol, kaempferol-3-*O*-rutinoside, naringenin-*C*-hexoside, hesperetin, apigenin-7-*O*-glucoside, quercetin, isorhamnetin-3-*O*-hexoside, apigenin-acetylglucosidase, isohamnetin-lutelion, and isorhamnetin-luteolin [18,21,22]. Phenolic terpenes found in rosemary include diterpenes such as carnosol, carnosic acid, rosmanol, epirosmanol, isorosmanol, rosemaridiphenol, rosmadial, Methoxycarnosol and Methoxycarnosate [18,21,22]. In addition, triterprnres such as betulinic acid, oleanolic acid, and ursolic acid (Table 1) [23,24].

**Figure 2:**
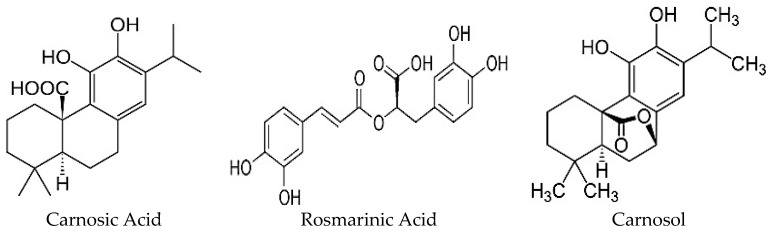
Structure of the major polyphenols of *Rosmarinus officinalis*: Carnosic acid, Rosmarinic acid and Carnosol.

The polyphenols found in highest quantity in RE are carnosic acid (CA), carnosol (COH) and rosmarinic acid (RA) (Figure 2) [22,25] and their production is influenced by growth conditions such as sunlight exposure, soil quality, and water availability [10]. Furthermore, anatomical regions of this herbal plant have varying levels of total phenolic content whereby the leaves contain the highest concentration of polyphenols in comparison to stem, branch and flower regions [26]. On the other hand, the choice of solvent and extraction method affects the chemical composition of the extract with the possibility of losing lipid soluble chemicals by an aqueous-based extraction method and water soluble chemicals by nonpolar solvent (ethanol, methanol)-based extraction. Regardless of the extraction method used, RE has been shown to have antioxidant, anti-inflammatory, anti-microbial, anti-tumorigenic, and anti-hyperglycemic properties. These biological effects are highly correlated with the polyphenolic content and CA, COH and RA are suggested to be responsible. The present review focuses on the existing studies examining the anti-diabetic effects of RE. Studies examining the anti-diabetic properties of CA, COH and RA are also included. In the following sections, in vitro and in vivo animal and human studies are presented.

## 3. Evidence of Anti-Diabetic Effects of Rosemary Extract: In Vitro Studies (Cell Free Models)

Digestive enzymes convert starch to maltose and iso-maltose which then travel to the small intestine where they are converted along with sucrose to monosaccharides (glucose and fructose) by α-glucosidase (sucrase and maltase) and transported by the intestinal sodium-glucose cotransporter thereby increasing blood glucose levels [27]. Inhibitors of α-glucosidase are used in the management of hyperglycemia present in T2DM. Acarbose is a currently prescribed anti-diabetic drug that reduces the rate of glucose absorption through inhibition of α-glucosidase in the brush border of the small intestine and pancreatic α-amylase found in the lumen of the small intestine [27]. RE exposure (5.5 mg/mL–55 mg/mL) was found to have significant α-glucosidase inhibitory activity (60% decrease) [28], while, in another study, the IC_50_ was found to be 683–711 μg/mL, making RE extract the most potent among 31 other extracts of herbs and spices tested [29] (Table 2). Furthermore, RA (8.88 mM) was also demonstrated to significantly inhibit porcine pancreatic amylase activity [30].

RE was found to be a potent inhibitor of DPP-IV (IC_50_ of 6.5 ± 0.4 μM gallic acid equivalents (GAE)) comparable to sitagliptin (0.05 ± 0.01 μM) [31]. Computational modeling experiments demonstrated that carnosol found in highest levels in RE had a strong binding affinity to DPP-IV [31].

The breakdown of triglycerides is regulated by lipases such as hormone-sensitive lipase (HSL) in adipose tissue and pancreatic lipase (PL) in the lumen of the small intestines. Excessive activation of hormone sensitive lipase leads to increased serum levels of free fatty acids (FFA) and insulin inhibits this hormone [32]. RE at concentrations of 6.3–200 µg/mL had a dose-dependent inhibitory activity ranging from 2.4–64.9% for HSL and 36.8–95.1% for PL effects that were less potent but in the same direction as orlistat, an FDA approved anti-obesity drug [33]. Pure RA was also shown to inhibit HSL and PL in a dose dependent manner although the degree of inhibition was lower than the whole extract [33]. Additionally, in vitro analysis indicated that CA enriched rosemary extract (100 μg/mL) inhibited PL activity by 70% [34]. COH, a major RE constituent, was found to have potent inhibitory activity (IC_50_ = 62.5 µM) against rat liver diacylglycerol acyltransferase 1 (DGAT1), an enzyme that plays a fundamental role in triglyceride synthesis [35]. COH (20 µM, 40 µM) was found to inhibit intracellular de novo triglyceride synthesis by 67.5–90.6% without affecting cell viability [35]. These studies clearly indicate that similar to the action of insulin, rosemary, CA and RA have antilipolytic activity in vitro.

## 4. Evidence of Anti-Diabetic Effects of Rosemary Extract: In Vitro Studies (Hepatocytes)

In vitro studies have shown that RE (100 μg/mL) has an insulin-like effect to inhibit the production of glucose by hepatocytes. RE was shown to significantly suppress gluconeogenesis in HepG2 hepatocytes (Table 3) [36]. In another study, RE (2.0–50 μg/mL) was shown to significantly increase hepatocyte (HepG2) glucose consumption in a dose-dependent manner (6% to 21%) [37]. In addition, a significant decrease in cellular glycogen content and increase glycolytic rate was seen [37]. CA (10–20 mM), a major polyphenolic constituent of RE, prevented palmitate-induced lipid accumulation in hepatocytes [38] indicating that CA may potentially block cellular lipid accumulation in the long term and help against insulin resistance and T2DM. Furthermore, it was found that treatment of HepG2 hepatocytes with COH (20–40 μM) resulted in inhibition of intracellular triglyceride synthesis via an effect that was associated with significant inhibition of diacylglycerol acyltransferase (DGAT1), the enzyme that plays a pivotal role in catalyzing the formation of triglycerides from Acetyl-CoA and diacylglycerol [35]. It is important to note that inhibition of DAGT1 has been proposed as a target approach for T2DM and obesity treatment. RE’s polyphenolic constituents may also provide protection against chemically-induced reactive oxygen species production and hepatocyte death. RA exhibited significant cytoprotective effects against mycotoxin-induced reactive oxygen species (ROS) production and induction of apoptosis by blocking effects on caspase-3 activation in hepatocytes [39].

## 5. Evidence of Anti-Diabetic Effects of Rosemary Extract: In Vitro Studies (Adipocytes)

Treatment of 3T3-L1 adipocytes with CA (3 μM) and COH (3 μM) inhibited the differentiation of 3T3-L1 pre-adipocytes into mature adipocytes and increased intracellular glutathione (GSH), an important antioxidant that prevents against ROS-induced damage (Table 4) [40]. Similarly, rosemary extract and CA were demonstrated to inhibit 3T3-L1 adipocyte differentiation, in part through inhibition of the transcription factor PPARγ [41] (Table 4). An increase in glucose uptake and intracellular lipid levels was seen in 3T3-L1 adipocytes treated with RE (50 µg/mL) (Table 4) [42].

In contrast, treatment with CA (0.1–10 µM) resulted in reduced intracellular lipid accumulation (19.1–33.4%), TG content (15.5–39.8%) and glycerol-3-phosphate dehydrogenase (GPDH) activity in 3T3-L1 adipocytes [43]. The activity of cytosolic GPDH, a key regulatory enzyme involved in triglyceride synthesis, dose-dependently decreased in CA treated adipocytes [43].

Corroborating with the above findings, CA significantly decreased mRNA expression of PPARγ, C/EBPα and SREBP1 indicating that it affects PPARγ/SREBP1 medicated adipogenesis [43]. Interestingly, CA treated adipocytes had a lower monounsaturated fatty acid (MUFA) to saturated fatty acid (SFA) ratio compared to control cells which was associated with a reduction of both mRNA and protein expression levels of stearoyl-CoA desaturase 1 (SCD1), a PPARγ dependent enzyme responsible for conversion of SFA to MUFA. MUFAs function as the major substrates for the synthesis of triglycerides and cellular membrane phospholipids in adipocytes, and an increased ratio of MUFA: SFA is correlated with de novo lipogenesis (Table 4) [43].

Carnosic acid treatment (1–20 uM) prevented LPS-stimulated elevations in the mRNA expression of tumor necrosis factor-α (TNFα), interleukin-6 (IL-6), and monocyte chemoattractant protein-1 (MCP-1) indicating a significant reduction in the inflammatory response of adipocytes [44].

Together, these findings suggest effects of RE and RE polyphenols on adipocyte lipid accumulation and anti-inflammatory effects.

## 6. Evidence of Anti-Diabetic Effects of Rosemary Extract: In Vitro Studies (Skeletal Muscle Cells)

Skeletal muscle tissue is important target of insulin and accounts for approximately 80% of insulin mediated glucose uptake in the postprandial state. As a consequence, it plays a predominant role in glucose homeostasis. Treatment of L6 myotubes with RE increased glucose uptake in a dose and time dependent manner [45]. Maximum stimulation seen with 5 µg/mL of RE was comparable to maximum stimulation seen with insulin (100 nM) and metformin (2 mM) (Table 5) [45].

In addition, treatment of L6 myotubes with CA (20 µg/mL for 6 h) resulted in significant increase in glucose uptake [46]. Furthermore, in another study by Naimi et al., 2016, CA significantly increased the glucose uptake in a dose- and time-dependent manner with concentration as low as 2 μM CA [47]. As proof-of-concept, the studies suggest that RE and its components increase skeletal muscle glucose uptake (rate-limiting step in skeletal muscle glucose metabolism) although other potential effects downstream of glucose uptake in this tissue remain to be explored.

All of the above in vitro studies indicate that RE has the potential to affect key insulin target tissues (liver, fat, and muscle) and directly induce anti-diabetic effects.

## 7. Evidence of Anti-Diabetic Effects of Rosemary Extract: In Vivo Animal Studies

Anti-hyperglycemic effects of rosemary and its components have been evaluated in different animal models of T2DM. Experimental interventions such as chemical (streptozotocin and alloxan) and dietary (high fat and high fructose diets) modifications have been employed to induce T2DM in animal models to study the effects of rosemary on associated biomarkers. Due to the lack of standardized pharmacological and clinical evidence, the formulations and doses of rosemary and its components used in these animal studies are variable. Notably, rosemary has not been extensively evaluated in clinical trials involving humans. Collectively, the current in vivo models demonstrate that rosemary and its main components CA and RA possess significant in vivo anti-hyperglycemic and anti-hyperlipidemic properties as outlined below.

## 8. Streptozotocin (STZ)-Induced Diabetes Model

Streptozotocin (STZ) is traditionally used to induce both type 1 and type 2 diabetes mellitus by promoting β-cell death through alkylation of DNA [48]. While a high dose of STZ potently impairs insulin secretion mimicking type 1 diabetes, low doses are known to induce a mild impairment of insulin secretion resembling the clinical features of T2DM. The reported studies reciprocally utilize low dose STZ to study the acute and chronic effects of rosemary and its polyphenolic constituents.

In streptozotocin (STZ)-induced diabetic mice, administration of an aqueous RE (10 g/L) for three months resulted in significant decrease in fasting plasma glucose (FPG) level (Table 6) [49]. There were no changes in alkaline phosphatase activity, bilirubin, and creatine indicating that rosemary did not cause cytotoxicity and histotoxicity [49]. In another study, administration of 50% ethanol extract of rosemary (20 mg/kg/day) in STZ rats significantly decreased plasma glucose levels due to inhibition of intestinal glucosidase enzyme activity [29]. In a more recent study, daily administration of aqueous RE (200 mg/kg/day) for three weeks caused significant reductions in blood glucose levels of both normal and STZ-induced diabetic rats [50]. These effects were associated with increased serum catalase (CAT), superoxide dismutase (SOD), glutathione peroxidase (GPx), vitamin C and vitamin G, while decreased lipid peroxidation marker MDA levels [50]. Similar effects were seen on blood glucose levels in STZ-induced diabetic rats given aqueous RE (1.11 g/mL) for four weeks [51]. In addition, the extract significantly reduced plasmatic FPG, TG, TC and low density lipoprotein (LDL) while increasing high density lipoprotein (HDL) and erythrocytes levels [51]. In agreement with the above studies, the fasting plasma blood glucose levels as well as TC, LDL and TG levels were significantly reduced while HDL was increased in STZ-induced diabetic rats given aqueous RE (200 mg/kg/day) two weeks before and three weeks after STZ injection [52]. Administration of aqueous RE (200 mg/kg/day) for 21 days in STZ-induced diabetic rats resulted in decrease in FPG, TC and total antioxidant capacity [53]. Furthermore, administration of aqueous RE (200 mg/kg/day) for four weeks prior and to streptozotocin injection in rats significantly protected against STZ-induced elevations in blood glucose levels which was correlated with a significant protection against pancreatic β-cell loss [54] In addition, RE increased serum insulin, C-peptide while decreased alanine aminotransferase (ALT) and aspartate aminotransferase (AST). Administration of dried rosemary leaves (5 g/100 g of diet) for approximately six weeks in STZ-induced diabetic and healthy Sprague-Dawley rats decreased the FPG, glycated hemoglobin (HbA1c), TC and TG and LDL levels in STZ- induced rats without effecting the plasma glucose levels or the lipid profile in the control group [55]. Oral administration of RE (200 mg/kg/day) with or without moderate intensity exercise training for eight weeks resulted in reduction of FPG and increased serum insulin levels in STZ-induced diabetic rats [56]. These effects were associated with an increased antioxidant activity including increase in serum CAT, SOD and GPx while decreasing malondialdehyde (MDA) [56]. In agreement, a more recent study showed that RA (120–200 mg/kg) dose-dependently decreased plasma glucose levels, and improved insulin sensitivity as supported by HOMA-IR analysis [57]. In STZ and high fat diet (HFD) induced diabetic rats, RA (577 µg/mL) decreased FPG levels and increased insulin levels without affecting liver glycogen levels [58]. These effects were associated with inhibition of intestinal brush border membrane levels of SGLT1 by 50%, an active glucose transporter responsible for glucose absorption into the bloodstream [58]. Oral administration of RE (200 mg/kg/day) for six weeks protected against the reduced antioxidant status of diabetic rats with decreased GSH, CAT, and SOD; and increased lipid peroxidation marker MDA levels [59]. Assessment of renal biomarkers including serum creatinine, uric acid and urea levels showed that RE had renoprotective effects (Table 6) [59].

## 9. Alloxan-Induced Diabetes Model

In addition to the STZ-induced diabetic model, the alloxan-induced diabetes animal model is also used extensively. Alloxan causes diabetes by rapid depletion of pancreatic β-cells leading to inflammation and sustained hyperglycemia secondary to a reduction in insulin release into circulation. In alloxan-induced diabetic rabbits, ethanol RE (200 mg/kg) for one week led to a significant reduction in FPG and increase in insulin levels (Table 6) [60]. In contrast, another study indicated that intramuscular administration of volatile oil of RE (25 mg/kg) for 30, 60 and 120 min inhibited insulin release and increased blood glucose levels leading to hyperglycemia in normal and alloxan-induced diabetic rabbits [61]. In another study, oral administration of powdered RE added as 20% of diet or 20% aqueous RE for 45 days to alloxan-induced diabetic rats significantly decreased the FPG levels compared to control [62]. In addition, the treatment demonstrated to reduce alloxan-induced hepatocyte vacuolar degeneration, necrosis, small hemorrhages and dilatation of hepatic sinusoids indicating hepatoprotective effects [62]. Moreover, administration of RA (100–200 mg/kg) to alloxan-induced diabetic rats for eight weeks significantly inhibited glomerular hypertrophy, glomerular number loss and glomerulosclerosis compared with diabetic control indicating RA’s renoprotective properties [63]. Administration of 250 and 500 mg/kg/day of RE mixed with water (70%) for 63 days in male rats did not affect serum blood glucose, TG, TC levels or body weight but testosterone levels, spermatogenesis, sperm density and motility were significantly decreased [64]. In addition, alanine aminotransferase (ALT) and aspartate aminotransferase (AST), enzymes released due to liver damage, were also decreased (Table 6).

## 10. Genetically-Induced Diabetes Models

Oral administration of CA (approximately 17 mg/kg/day) to obese leptin receptor deficient mice for five weeks resulted in significant protection against fat-induced fasting and non-fasting hyperglycemia, improved glucose tolerance as well as decreased serum insulin levels [65]. In addition, CA significantly inhibited weight gain, decreased regional areas of visceral fat, and prevented against fat accumulation in white adipose tissue and liver [65]. Moreover, animals supplemented with CA exhibited decreased serum levels of TG, TC, and ALT, as well as significantly decreased hepatic lipid storage [65]. In contrast, obese mice and their lean counterparts fed 0.5% ethanol extract of rosemary enriched with CA (40%) incorporated in their standard chow for 64 days did not show significant differences in their glucose levels compared to control rats, although circulating insulin levels were found to be significantly decreased only in the lean rats (Table 6) [66]. Noteworthy, the plasma glucose levels in all animals were within normal physiological range with a non-significant, slight increase in obese counterparts. The study also demonstrated a significant inhibition of gastric lipase (GL) in the stomach and pancreatic lipase (PL) in small intestine of rats consuming the RE [66].

## 11. Diet-Induced Diabetes Model

Apart from animal models of genetic and chemically-induced obesity and T2DM, the effects of RE have been examined in dietary animal models of obesity and T2DM. Daily, dietary supplementation of RE (500 mg/kg) standardized to contain 20% CA for 16 weeks in mice that were started on a high-fat diet (HFD) as juveniles significantly protected against HFD-induced elevations in plasma glucose and TC levels compared with HFD control mice (Table 6) [34]. Notably, fasting insulinemia remained low during the length of the study and no significant differences were observed between the groups. Correlating with the observed reductions in total cholesterol levels, HFD mice supplemented with RE displayed significant decreases in fat mass and one to twofold increase in total fecal lipid content compared to HFD-fed control mice [34]. Additionally, another study indicated that administration CA (20 mg/kg) in 5–20 mg/kg olive oil loaded mice, significantly repressed the elevation of TG levels, prevented epididymal fat gain and inhibited pancreatic lipase activity [67]. Administration of 200 mg/kg of RE for 50 days in mice fed high fat diet, resulted in reduced body weight (b.w) and fat mass and increase of fecal lipid excretion, while hepatic triglyceride content was decreased [68]. Similarly, daily administration of aqueous RE (100 mg/kg b.w) to high-cholesterol fed mice for 36 days resulted in significant decline in plasma TG, TC, LDL levels, while HDL levels were increased compared to control mice [69]. Furthermore, administration of aqueous RE (70–140 mg/kg b.w) and non-esterified phenolic RE (7–14 mg/kg b.w) for four weeks resulted in significant reduction in TC and non-HDL levels compared to control [70]. High-fructose fed mice given daily dose of RA (100 mg/kg b.w) for 60 days decreased fasting plasma glucose levels, improved glucose tolerance and reduced plasma insulin and glycated HbA1c levels (Table 6) [71].

In addition to T2DM, type 1 diabetes mellitus (T1DM), an autoimmune disorder, is characterized by pancreatic β-cell destruction and hyperglycemia. Very limited number of studies investigating the effects of RE and its polyphenols in T1DM exist. A study demonstrated that co-treatment of RA and anti-CD154 monoclonal antibody improved islet allograft survival in a murine model [72]. In addition, the co-treatment resulted in fewer apoptotic cells and increased expression of insulin and glucagon [72]. Pancreatic islet transplantation is an area of intense research in the field of T1DM and immunosuppressants are used to reduce transplant rejection, however, they have detrimental/debilitating side effects. This study indicated that RE polyphenols improved outcome and minimized side effects when used in islet transplantation, however, further research is required to investigate their effectiveness in T1DM.

## 12. Anti-Diabetic Effects of Rosemary Extract and Its Main Polyphenolic Constituents: In Vivo Human Studies

In addition to numerous in vitro and in vivo studies, RE has been recently investigated in humans. Participants were randomly selected into three groups and treated with 2, 5 or 10 g/day of dried rosemary leaf powder for eight weeks [73] (Table 7). Blood samples were taken from participants before and after the study. FPG was decreased by 18.25%, 15.74% and 11.2% in the 10, 5 and 2 g/day group, respectively. TC levels were significantly decreased by 34.48% in the 10 g/day group and 17.97% and 11.48% in the 5 g/day and 2 g/day groups, respectively. LDL cholesterol in this group was also significantly lowered by 32.28% in the 10 g/day treated group, and 28.46% and 15.58% in the 5 g/day and 2 g/day groups, respectively. Additionally, HDL cholesterol was increased by 22.91% in the 10 g/day group and 15.21% and 4.54% in the 5 g/day and 2 g/day groups, respectively. Furthermore, triglyceride levels were also decreased by 29.06% in the 10 g/day group and 21.3% and 14.97% in the 5 g/day and 2 g/day groups, respectively. In addition to the improvement in the overall lipid profile, rosemary powder seems to exhibit high antioxidant properties by decreasing MDA by 36.21%, 12.43% and 13.6% in the 10 g/day, 5 g/day and 2 g/day treated groups, respectively. Glutathione reductase (GR) was decreased by 15.36%, 6.73%, and 0.95% in the 10 g/day, 5 g/day and 2 g/day treated groups respectively. Vitamin C indicated an increase by 19.93%, 11.37% and 8% in the 10 g/day, 5 g/day and 2 g/day treated groups, respectively. In addition, β carotene levels increased by 45.23%, 33.33% and 7.5% in the 10 g/day, 5 g/day and 2 g/day treated group. Additionally, there have been a few studies indirectly examining the effects of rosemary supplements with non-conclusive results. An observational, prospective, monocenter study examined the effect of 21 days of oral supplementation of aqueous RE (containing 77.7 mg RE with 0.97 mg COH, 8.60 mg CA, and 10.30 mg RA) in twelve healthy young volunteers found a significant decrease in plasminogen activator-inhibitor-1 (PAI-1) levels suggesting that RE may have anti-inflammatory and anti-blood clotting activity in vivo [74]. Another observational study demonstrated that administration of RE and oleanolic acid (440 mg thrice a day for four weeks and additional 880 mg twice a day for four weeks) in patients with osteoarthritis, fibromyalgia, and rheumatoid arthritis was protective against inflammatory rheumatic diseases, particularly in those with initial serum c-reactive protein (CRP) levels >7.0 mg/L (Table 7) [75].

## 13. Conclusions

Rosemary extract and the rosemary extract polyphenols carnosic acid and rosmarinic acid have been shown to have insulin-like effects in insulin target cells in vitro and to exert significant anti-diabetic effects in different animal models of T2DM in vivo. Rosemary extract and rosemary extract polyphenols exhibit protective properties against hyperlipidemia and hyperglycemia in genetic, chemically-induced and dietary animal models of obesity and T2DM. These promising findings from in vivo animal studies suggest a potential to use RE and its polyphenolic constituents towards the management of blood glucose levels and diabetes. However, more studies are warranted to fill the gaps in research. On the one end of the research spectrum, in vitro studies are needed to delineate the direct effects of RE and its polyphenolic constituents in specific cell and tissue types and thereby ascertain their mechanism of action. On the other end, clinical trials are warranted to examine the effects of RE and its polyphenolic constituents directly in humans. Furthermore, more research is needed to address questions surrounding bioavailability of RE and its polyphenolic components, and therapeutic dosage ranges relevant in humans.

## Figures and Tables

**Table 1:** Polyphenolic profile of Rosemary extract (RE).

Polyphenols						
Phenolic Acids			Flavonoids		Phenolic Terpenes	
Hydroxycinnamic Acids	Hydroxybenzoic Acids	Hydroxyphenylacetic Acids	Flavones	Flavonols	Diterpenes	Triterprnres
Rosmarinic acid (C_18_H_16_O_8_) [18]	Vanillic acid (C_8_H_18_O_4_) [22]	Homovanillic acid (C_9_H_10_O_4_) [22]	Apigenin (C_27_H_30_O_15_) [18]	Rutin (C_27_H_30_O_16_) [18]	Carnosol (C_20_H_26_O_4_) [18,21,22]	Betulinic acid (C_30_H_48_O_3_) [23,24]
Chlorogenic acid (C_16_H_18_O_9_) [18,21,22]	Syringic acid (C_9_H_10_O_5_) [18,21,22]	p-Hydroxybenzoic acid (C_7_H_6_O_3_) [22]	Luteolin (C_15_H_10_O_6_) [18]	Kaempferol (C_15_H_10_O_6_) [18,21,22]	Carnosic acid (C_20_H_28_O_4_) [18,21,22]	Oleanolic acid (C_30_H_48_O_3_) [23,24]
o-,m-,p-Coumaric acid (C_9_H_8_O_3_) [18,21,22]	Caffeic acid (C_9_H_7_O_4_) [18,21,22]		Hispidulin (C_16_H_12_O_6_) [23,24]	Kaempferol-3-*O*-rutinoside (C_27_H_30_O_15_) [18,21,22]	Rosmanol (C_20_H_26_O_5_) [18,21,22]	Ursolic acid (C_30_H_48_O_3_) [23,24]
m-Hydroxybenzoic acid (C_7_H_6_O_3_) [18,21,22]	Protocatechuic acid (C_7_H_6_O_4_) [18,21,22]		Genkwanin (C_16_H_12_O_5_) [23,24]	Naringenin-*C*-hexoside (C_21_H_22_O_10_) [21,22]	Epirosmanol (C_20_H_26_O_5_) [21,22]	
Coumaroylqunic acid (C_16_H_18_O_8_) [21,22]	Dicaffeoylquinic acid (C_25_H_24_O_12_) [18,21,22]			Hesperetin (C_28_H_34_O_15_) [18]	Isorosmanol (C_20_H_26_O_5_) [18,21,22]	
Ferulic acid (C_16_H_20_O_9_) [21,22]				Apigenin-7-*O*-glucoside (C_21_H_20_O_10_) [18]	Rosmaridiphenol (C_20_H_28_O_3_) [18]	
				Quercetin (C_15_H_10_O_7_) [18]	Rosmadial (C_20_H_24_O_5_) [18]	
				Isorhamnetin-3-*O*-hexoside (C_22_H_22_O_12_) [23,24]	Methoxycarnosol [23,24]	
				Apigenin-acetylglucosidase [23,24]	Methoxycarnosate [23,24]	
				Isohamnetin-lutelion [23,24]		
				Isorhamnetin-luteolin [23,24]		

**Table 2:** Anti-diabetic Effects of Rosemary Extract and its Main Polyphenolic Constituents: cell-free model studies.

Cell-Free Model	Dose	Effects	Reference
α-glycosidase	RE 5.5–55 mg/mL	↓ α-glycosidase activity (60%)	[28]
α-glycosidase	50% ethanolic RE (IC_50_: 83–711 µg/mL)	↓ α-glucosidase	[29]
Porcine Pancreatic α-amylase (PPAM)	RA 8.88 mM	↓ PPAM activity (85%)	[30]
Dipeptidyl Peptidase IV (DPP-IV); Protein Tyrosine Phosphatase 1B (PTP1B)	Methanolic RE (IC_50_ = 6.5 ± 0.4 µM) RA (IC_50_ = 14.1 ± 1.7 µM) COH (IC_50_ > 100 µM)	↓ DPP-IV activity (50%) RE (↓ PTP1B 40.9 ± 7.2%).	[31]
Porcine pancreatic lipase (PL); Hormone sensitive lipase (HSL)	Methanolic RE 6.3–200 µg/mL RE (IC_50_ = 13.8 for PL and 95.2 µg/mL for HSL) RA (IC_50_ = 125.2 for PL and 51.5 µg/mL for HSL)	↓ PL and HSL activity RE (↓ PL: 36.8–95.1%) RE (↓ HSL: 2.4–64.9%) RE > RA	[33]
Human PL	Acetone RE 100 µg/mL rich in CA	↓ human PL (70%)	[34]
Rat liver diacylglycerol acyltransferase (DGAT1)	COH (IC_50_ = 62.5 ± 2.1 µM)	↓ DGAT1 activity (67.5–90.6%)	[35]

**Table 3:** Anti-diabetic Effects of Rosemary Extract and its Main Polyphenolic Constituents: in vitro studies (hepatocytes).

Cell/Model	Treatment	Effects	Reference
HepG2 hepatocytes	Methanolic RE 100 μg/mL	↓ gluconeogenesis	[36]
HepG2 hepatocytes	Methanolic RE 0.4, 2, 10, 50 µg/mL	↑ glucose consumption	[37]
		↑ glycolytic rate	
		↓ glycogenesis comparable to metformin	
		↑ β-oxidation	
		↓ decreased fatty acid synthesis	
		↔ cell viability	
HepG2 hepatocytes	CA 10–20 µM	↓ palmitate-induced lipid accumulation	[36]
		↔ cell viability	
HepG2 hepatocytes	COH 20–40 µM	↓ de novo formation of intracellular TG	[35]
		↔ cell viability	
HepG2 hepatocytes	RA 25–50 µM	↓ apoptosis	[39]
		↓ ROS production	

**Table 4:** Anti-diabetic Effects of Rosemary Extract and its Main Polyphenolic Constituents: in vitro studies (adipocytes).

Cell	Treatment	Effects	Reference
3T3-L1 adipocytes	CA 3 µM, COH 3 µM	↓ differentiation	[40]
		↑ intracellular GSH	
3T3-L1 adipocytes	Acetone RE 10–30 μg/mL CA 0.3–20 μM	Inhibited adipocyte differentiation ↔ cell viability	[41]
3T3-L1 adipocytes	RE 50 µg/mL	↑ intracellular lipid	[42]
		↑ glucose uptake	
3T3-L1 adipocytes	CA 0.1–10 µM	↓ intracellular lipid accumulation	[43]
		↓ TG content (15.5–39.8%)	
		↓ GPDH activity	
		↔ cell viability	
3T3-L1 adipocytes pretreated with LPS	CA 0–20 µM	↓ mRNA expression of TNFα, IL-6 and MCP-1	[44]
		↓ TLR4 protein expression	
		↓ Phospho-ERK levels	
		↓ NF-κB activation	

**Table 5:** Anti-diabetic Effects of Rosemary Extract and its Main Polyphenolic Constituents: in vitro studies (muscle cells).

Cell/Model	Treatment	Effects	Reference
L6 myotubes	Methanolic RE 0.1–50 µg/mL	↑ glucose uptake (GU) dose- and time-dependent Max stimulation: 5 µg/mL for 4 h comparable to insulin and metformin. ↔ cell viability	[45]
L6 myotubes	CA 1–50 µM	↑ GU in a dose- and time-dependent manner Max stimulation: 20 µM for 6 h comparable to insulin and metformin ↔ cell viability	[46]
L6 myotubes	Methanolic CA 0.1–10 µM	↑ GU dose- and time-dependent Max stimulation: 2 µM for 4 h comparable to insulin and metformin ↔ cell viability	[47]

**Table 6:** Anti-diabetic Effects of Rosemary Extract and its Main Polyphenolic Constituents: in vivo animal studies.

Animal Model	Dose	Glucose	Other Measures	Reference
Streptozotocin (STZ)-Induced Diabetic Model				
STZ-induced diabetic Swiss albino mice	Ad libitum (10 g leaves of rosemary in 1 L boiling water) for 3 months	↓ FPG in healthy and diabetic animals	↔ creatinine, urea bilirubin, total albumin, alkaline phosphatase	[49]
STZ-induced diabetic male	aqueous and ethanolic RE 20 mg/kg/day	↓ plasma glucose	↓ α-glucosidase (AGc)	[29]
ddY mice				
Male Wistar rats	RA 577 µg/mL as drinking fluid for 14 days	↓ FPG ↓ OGTT ↓ HOMA-IR indices ↑ serum insulin	↓ hepatic glycogen content	[58]
STZ-induced diabetic male albino rats	aqueous RE, 200 mg/kg/day for 3 weeks	↓ FPG	↑ vitamin C	[50]
STZ-induced diabetic male albino rats	aqueous RE 4 g/kg/day for 4 weeks	↓ FPG (20%)	↓ TC, TG, LDL ↑ HDL	[51]
STZ-induced diabetic male albino rats	aqueous RE, 200 mg/kg/day 2 weeks prior and 3 weeks after STZ	↓ FPG (36.9%)	↓ TC, TG, LDL ↑HDL ↑ hemoglobin	[52]
STZ-induced diabetic male albino rats	aqueous RE, 200 mg/kg/day for 21 days	↓ FPG	↓ TC ↓ TG ↑ TAC	[53]
STZ-induced diabetic male albino rats	aqueous RE, 200 mg/kg/day 2 weeks prior and 3 weeks after STZ	↓ FPG in both groups ↑ serum insulin ↑ C-peptide ↓ β-cell loss	↑ total albumin	[54]
STZ-induced diabetic male Dawley rats	Dried rosemary leaves powder 5 g/100 g of diet	↓ FPG (53.97%) ↓ HbA1c (24.56%)	↓ TG (45.43%) ↓ TC (39.31%) ↓ LDL (33.89%)	[55]
Male albino Wistar rats	aqueous RE 200 mg/kg/day with/without moderate intensity exercise training for 8 weeks	↓ FPG ↑ Serum CAT, SOD, GPx ↑ serum insulin ↓ MDA		[56]
Sprague-Dawley male albino rats	aqueous RE 200 mg/kg/day for 6 weeks	↓ FPG	↑ Serum CAT, SOD, GSH ↓ MDA ↓ urea, uric acid and creatinine levels	[59]
Male Wistar rats	Intraperitoneal injection of 120, 160, 200 mg/kg RA for 7 days (acute) and 28 days (chronic)	↓ FPG ↓ OGTT ↓ HOMA-IR indices ↑ ITT Normalized serum insulin	↓ hepatic PEPCK expression/gluconeogenesis	[57]
Alloxan-Induced Diabetes Model				
Alloxan-induced diabetic rabbits	ethanol RE, 200 mg/kg for 6 h (acute); for 1 week (subacute)	↓ FPG in healthy and diabetic rabbits ↑ plasma insulin	↓ MDA ↑ SOD ↑ CAT	[60]
Alloxan-induced male diabetic rabbits	volatile RE, 25 mg/kg intramuscular injection for 30, 60 and 120 min	↑ serum glucose		[61]
		↓ serum insulin		
Alloxan-induced Sprague-Dawley male albino rats	20% aqueous RE and 20% RE powdered food for 45 days	↓ FPG	↓ hepatocyte necrosis ↓ small hemorrhages ↓ hepatocyte degradation	[62]
Alloxan-induced Sprague-Dawley uninephrectomized rats	RA 100–200 mg/kg/day for 8 weeks		↓ glomerulosclerosis ↓ creatinine and urea ↓ glomerular number ↓ serum MDA	[63]
Male adult Sprague-Dawley rats	70% aqueous RE, 250 and 500 mg/kg/day for 63 days	↔ serum glucose	↔ body weight TG, TC ↓ alanine aminotransferase (ALT) ↓ Aspartate Aminotransferase (AST) ↓ spermatogenesis ↓ testosterone ↓ sperm motility	[64]
Genetically-Induced Diabetes Models				
Male ob/ob mice	CA 17 mg/kg/day for 5 weeks	↓ FPG (18%) ↓ OGTT glucose ↓ serum insulin (47%)	↓ TC (24%) ↓ TG (60%) ↓ plasma FFA (13%) ↓ hepatic lipids ↓ ALT (64%)	[65]
Female Zucker lean (fa/+) and obese (fa/fa) rats	0.5% w/w of aqueous RE enriched with CA for 64 days	↔ plasma glucose ↓ insulin levels in lean animals	Inhibited gastric lipase activity in both lean (70%) and obese animals (80%) ↓ Serum total cholesterol, TG, LDL ↑ HDL	[66]
Diet-Induced Diabetes Models				
HFD-treated male C57BL/6J mice	aqueous RE, containing 20% CA 500 mg/kg/day for 16 weeks	↓ FPG (72%) ↔ insulin	↓ body weight ↑ fecal total lipid content (1–2 fold) ↓ fat mass ↓ TC (68%) ↔ TG	[34]
HFD- (olive oil) treated male ddY mice	CA 20 mg/kg for 14 days COH 200 mg/kg for 14 days		↓ body weight (7%) ↑ epididymal fat ↓ pancreatic lipase (IC50 12 and 4.4 μg/mL for CA and COH respecively)	[67]
HFD-treated male C57BL/6J mice	ethanolic RE 20 or 200 mg/kg/day for 50 days	↔ FPG ↔ glucose tolerance ↔ insulin	↓ body weight and fat mass (64% and 57%) ↓ Hepatic TG (39%) ↔ serum TG and TC ↑ fecal lipid excretion	[68]
Diet-induced HC female BALB/c mice	aqueous RE, 100 mg/kg/day for 36 days		↓ TC, TG, LDL ↑ HD	[69]
Diet-induced HC Wistar rats	aqueous RE, aqueous 70–140 mg/kg/day RE non-esterified phenolic 7–14 mg/kg/day of for 4 weeks		↓ TC (39.8%) ↓ non-HDL (44.4%)	[70]
Fructose-fed Swiss albino mice	RA 100 mg/kg/day for 60 days	↓ FPG levels ↓ HbA1c ↓ OGTT glucose ↓ plasma insulin	↑ diaphragm glucose utilization	[71]

**Table 7:** Anti-diabetic Effects of Rosemary Extract and its Main Polyphenolic Constituents: in vivo human studies.

Study Methodology	Treatment	Effect	Reference
48 healthy individuals	Dry rosemary powder 2, 5 or 10 g/day, for 8 weeks	↓ FPG ↓ TC, ↓ LDL, ↓ TG, ↑ HDL ↓ MDA, ↓ GR ↑ vitamin C ↑ β-carotene	[73]
12 healthy, young volunteers	RE 77.7 mg	↓ PAI-1	[74]
	COH 0.97 mg		
	CA 8.6 mg		
	RA 10.30 mg for 21 days		
72 patients with rheumatic disease including osteoarthritis (OA), rheumatoid arthritis, fibromyalgia (FM)	Meta050 compound (RE, oleanolic acid and reduced iso-alpha-acids)	↓ CRP	[75]
	440 mg/day for 4 weeks 3 times per day	↓ arthritis pain scores	
	880 mg/day for 4 weeks 2 times per day	↔ fibromyalgia scores	

## Abbreviations

ALP	Alkaline phosphatase
ALT	Alanine aminotransferase
AST	Aspartate transaminase
CA	Carnosic acid
CAT	Catalase
C/EBPα	CCAAT-enhancer binding proteins,
COH	Carnosol
CRP	C-reactive protein
DDP-4	Dipeptidyl peptide 4
DGAT1	Diacylglycerol acyltransferase 1
ERK	Extracellular signal-regulated kinase
FDA	Food and Drug Administration
FFA	Free fatty acids
FM	Fibromyalgia
FPG	Fasting plasma glucose
GL	Gastric lipase
GLP-1	Glucagon-like peptide 1
GPDH	Glycerol-3-phosphate dehydrogenase
GPx	Glutathione peroxidase
GSH	Glutathione
GU	Glucose uptake
HSL	Hormone sensitive lipase
HDL	High-density lipoprotein
HFD	High-fat diet
HbA1c	Glycated hemoglobin
IDF	International Diabetes Federation
ITT	Insulin tolerance test
LDL	Low-density-lipoprotein
MCP-1	Monocyte chemoattract protein 1
MDA	Malondialdehyde
MUFA	Monounsaturated fatty acids
NF-kB	Nuclear factor kappa-light-chain-enhancer of activated B cells
OA	Osteoarthritis
OGTT	Oral glucose tolerance test
PAI-1	Plasminogen activator inhibitor-1
PEPCK	Phosphoenolypyruvate carboxykinase
PL	Pancreatic lipase
PPAR	Peroxisome proliferator activated receptor,
RA	Rosmarinic acid
RE	Rosemary extract
ROS	Reactive oxygen species
SCD1	Stearoyl coA desaturase 1
SFA	Saturated fatty acids
SGLT1	Sodium glucose cotransporters 1
SGLT2	Sodium-glucose cotransporter 2,
SOD	Superoxide dismutase
SREBP1	Sterol regulatory element binding protein 1
STZ	Streptozotocin
T1DM	Type 1 diabetes mellitus
T2DM	Type 2 diabetes mellitus
TC	Total cholesterol
TG	Triglycerides
TNFα	Tumor necrosis factor α
